# Importance of Vitamin Supplementation During Pregnancy: Pancytopenia in a 3-Month-Old Neonate

**DOI:** 10.3390/reports9020151

**Published:** 2026-05-15

**Authors:** Cathérine Van Den Plas, Toon van Genechten, Marie-Berthe Maes, Kathleen Deiteren, Catharina van der Heijden

**Affiliations:** 1 Resident Clinical Biology, University of Antwerp, Drie Eikenstraat 655, 2650 Edegem, Belgium; 2 Paediatrics Department, University Hospital of Antwerp, Drie Eikenstraat 655, 2650 Edegem, Belgium; toon.vangenechten@uza.be; 3 Laboratory Medicine, University Hospital of Antwerp, Drie Eikenstraat 655, 2650 Edegem, Belgium; marie-berthe.maes@uza.be (M.-B.M.); kathleen.deiteren@uza.be (K.D.); catharina.vanderheijden@uza.be (C.v.d.H.)

**Keywords:** vitamin B12 deficiency, pancytopenia, celiac disease

## Abstract

**Background and Clinical Significance**: Vitamin B12 deficiency in infancy is an uncommon but reversible cause of severe hematologic abnormalities and potential neurologic injury, particularly in exclusively breastfed infants whose vitamin B12 status depends on maternal stores. Because its clinical presentation may mimic bone marrow failure syndromes or hematologic malignancies, diagnosis can be challenging and delayed; **Case Presentation**: We report a case of early infantile pancytopenia ultimately attributed to profound vitamin B12 deficiency secondary to maternal celiac disease. Prompt recognition and treatment with cobalamin supplementation resulted in rapid hematologic recovery and a favorable clinical outcome; **Conclusions**: This case underscores the importance of considering vitamin B12 deficiency in the differential diagnosis of unexplained cytopenias in infants and highlights the critical role of maternal nutritional status in neonatal health. Improved awareness and targeted screening of at-risk mothers during pregnancy and lactation may prevent severe but readily treatable complications in affected infants.

## 1. Introduction and Clinical Significance

Vitamin B12 (cobalamin) deficiency in infancy is a rare but treatable cause of failure to thrive and neurologic developmental delay. Clinical manifestations of vitamin B12 deficiency generally appear within the first few months of life and may include developmental delay or regression, irritability, feeding difficulties, seizures, muscular hypotonia, anemia, and microcephaly. If the condition is not recognized and treated promptly with vitamin B12 supplementation, irreversible neurological damage may occur [1].

Infants are particularly vulnerable due to limited hepatic cobalamin stores at birth and complete dependence on maternal vitamin B12 status, especially when they are exclusively breastfed [1,2,3]. Maternal conditions associated with reduced vitamin B12 uptake are a strict vegetarian diet, pernicious anemia, or other malabsorptive disorders, such as celiac disease [4,5,6]. In newly diagnosed untreated adult patients with celiac disease, vitamin B12 deficiency was reported in 19% of cases [7]. Although pregnancy-specific prevalence data in women with celiac disease remain limited, this population is considered at increased risk because of impaired intestinal absorption combined with the increased maternal vitamin requirements during gestation [8].

The most common hematologic manifestation of vitamin B12 deficiency is macrocytic anemia; however, in severe cases, vitamin B12 deficiency can even manifest as pancytopenia or microangiopathic hemolytic anemia (MAHA). When present in infants, pancytopenia poses a significant diagnostic challenge, raising concern for bone marrow failure syndromes, clonal hematologic disease, or leukemia, often delaying diagnosis [9,10,11,12]. Early recognition of vitamin B12 deficiency during pregnancy and in infancy is essential, as it is associated with adverse maternal and infant health outcomes. Early treatment is crucial since it leads to rapid hematologic recovery and prevents irreversible neurological damage [13,14,15,16].

We present a case of a 3-month-old infant with pancytopenia and growth restriction as the sole symptoms of a severe vitamin B12 deficiency, highlighting the importance of maternal clinical evaluation and nutrition status for early recognition and diagnosis.

## 2. Case Presentation

A 3-month-old male infant was referred to the emergency department for failure to thrive and extreme pallor. Upon physical examination, the infant showed no signs or symptoms of acute distress or fever. No hepatosplenomegaly or lymphadenopathy was noted, and thorough neurodevelopmental assessment was consistent with his postnatal age. However, significant growth restriction was observed, with a body weight of 5.80 kg, corresponding to the 9th percentile for age.

Medical history revealed that he was born at term with a normal birth weight of 3.240 kg. He was exclusively breastfed. Maternal dietary history was reported as normal, and she had been taking folic acid regularly during pregnancy. Familial history for thalassemia or other inherited disorders was negative.

Initial laboratory investigations showed pancytopenia with a hemoglobin level of 3.9 g/dL and a normal mean corpuscular volume (MCV) of 96 fL, a platelet count of 18 × 10^9^/L, a leucocyte count of 6.34 × 10^9^/L, and a neutrophil count of 1.22 × 10^9^/L. Leucocyte and neutrophil counts further decreased to a level of 3.46 × 10^9^/L and 0.15 × 10^9^/L, respectively (see Table 1). Peripheral blood smear examination showed pronounced poikilocytosis, in particular teardrop cells and fragmentocytes. Lactate dehydrogenase (LDH) was 1874 U/L, liver function tests showed a total bilirubin of 5.5 mg/dL and indirect bilirubin of 5.1 mg/dL, indicating possible hemolysis. The iron profile of the patient showed no abnormalities. Additional testing included bone marrow biopsy, glucose-6-phosphate dehydrogenase (G6PD), hemoglobin electrophoresis, infectious serology for Parvovirus B19, as well as cytomegalovirus and Epstein–Barr virus, and PCR for Human Herpesvirus type 6 (HHV6).

**Figure 1:**
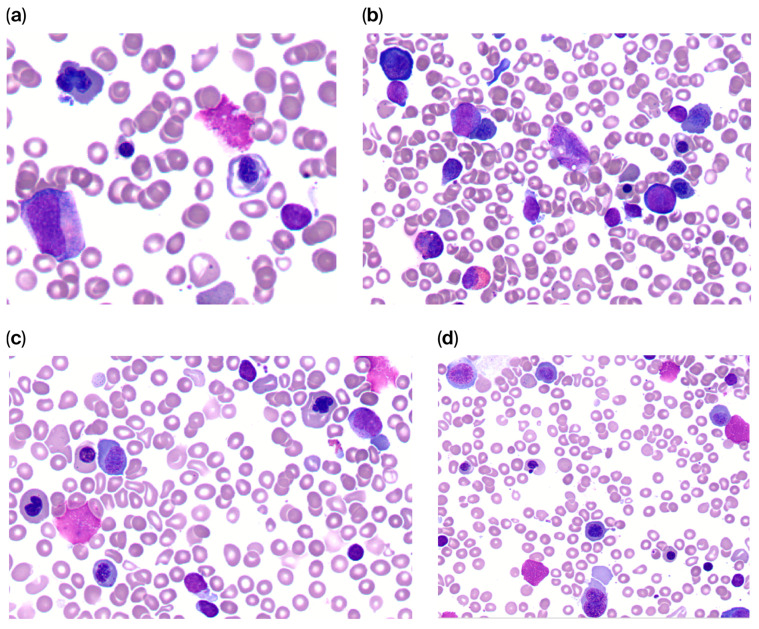
(**a**,**b**) (×100), (**c**,**d**) (×50): Bone marrow biopsies with morphologic features of dyserythropoiesis demonstrating nuclear budding (abnormal nuclear segmentation) and aberrant chromatin patterns in erythroid precursors.

The bone marrow biopsy demonstrated a normocellular bone marrow and dysplastic changes involving all three hematopoietic lineages, in particular an extreme dyserythropoiesis with pronounced megaloblastoid changes and nuclear abnormalities (Figure 1a–d).

These findings led to the hypothesis of a possible vitamin B12 or folic acid deficiency with a myelodysplastic neoplasm of childhood or bone marrow failure syndrome in the differential. Acute myeloid leukemia was initially considered; however, flow cytometric analysis demonstrated only a mildly increased blast population, consisting of a combination of myeloblasts and hematogones. Vitamin B12 was deficient with a level of 132 ng/L (reference value 211–911 ng/L), and the folic acid level was 15.4 µg/L (reference value > 3 µg/L). To definitively exclude myelodysplastic neoplasm or an inherited bone marrow failure syndrome, additional diagnostic testing was performed, including next-generation sequencing (NGS) for myeloid malignancies and whole exome sequencing, as well as diepoxybutane (DEB) testing and telomere length assessment for bone marrow failure.

A complete blood count test in the mother revealed a macrocytic anemia, with a high red blood cell MCV of 120 fL and hemoglobin level of 10.7 g/dL. Folic acid was within the range of normal, but vitamin B12 was deficient, with a level of 78 pg/mL. Upon further questioning, it was found that the mother suffered from celiac disease.

The patient was treated with an intramuscular injection of 100 mcg cobalamin and received transfusions of packed red blood cells and platelets. After ten days of inpatient management with continuous clinical and laboratory monitoring, the patient was discharged in good clinical condition following an additional intramuscular administration of 100 mcg cyanocobalamin. Further supplementation was not required, as breastfeeding was discontinued and the patient was transitioned to formula feeding. The option of continuing breastfeeding with maternal vitamin B12 supplementation was discussed with the mother; however, she elected to discontinue breastfeeding in consultation with the pediatrician and preferred to proceed with formula feeding.

Catch-up growth was observed, with the patient’s weight reaching 10.20 kg at seven months of age (60th percentile). The complete blood count completely recovered within four months after starting treatment (Table 1). Follow-up bone marrow biopsy showed a regenerative bone marrow without megaloblastic changes and nuclear abnormalities in the erythroid lineage. Additional molecular and genetic testing was all within normal range. Neurological development remained normal throughout follow-up, with age-appropriate developmental milestones consistently achieved at the expected time points.

## 3. Discussion

This case of severe infantile pancytopenia due to vitamin B12 deficiency highlights the crucial role of maternal micronutrient status in neonatal health and emphasizes the importance of comprehensive prenatal follow-up. Infants are entirely dependent on maternal vitamin B12 stores, particularly during exclusive breastfeeding, as neonatal hepatic cobalamin reserves are limited at birth. Consequently, unrecognized maternal deficiency can rapidly result in significant hematologic abnormalities and, if untreated, irreversible neurologic injury in the infant.

Preventive micronutrient supplementation during pregnancy is an essential public health measure to reduce adverse maternal and fetal outcomes. Among these, folic acid supplementation is universally recommended before conception and throughout early pregnancy due to its important role in the prevention of neural tube defects [17,18,19]. However, folic acid supplementation alone does not address coexisting vitamin B12 deficiency, which may remain undetected during pregnancy. In such cases, folic acid can normalize or partially correct hematologic findings while masking underlying cobalamin deficiency and allowing neurologic complications to progress [20,21,22].

While folic acid supplementation remains essential and should not be discouraged, exclusive reliance on folate without consideration of maternal vitamin B12 status may be insufficient, particularly in populations at increased risk of deficiency, including women with malabsorptive disorders, restrictive diets, or gastrointestinal disease. This risk is further amplified during pregnancy and lactation, when maternal vitamin B12 requirements are increased and deficiency may directly affect the exclusively breastfed infant [1,3,8].

Despite its essential role in DNA synthesis, erythropoiesis, and neurologic development, vitamin B12 is not routinely screened for or supplemented in many prenatal care programs. Maternal deficiency has been associated with adverse pregnancy outcomes such as low birth weight, intrauterine growth restriction, and preterm birth, and infants born to untreated mothers are at risk for severe hematologic manifestations and neurodevelopmental delays during infancy [11,18,23,24]. In rare cases, vitamin B12 deficiency can present with pancytopenia and bone marrow dysplasia, mimicking acute myeloid leukemia or inherited bone marrow failure syndromes and potentially leading to a diagnostic delay [6,9,25].

Several case reports have described severe vitamin B12 deficiency presenting with pancytopenia in early infancy, most commonly in exclusively breastfed infants of mothers with unrecognized nutritional deficiency states. Reported cases [2,4,5,9,10,12] typically present patients between 7 and 10 months of age and frequently demonstrate a combination of hematological and neurological manifestations, including muscular hypotonia, developmental delay, irritability, seizures, paleness, bruising, epistaxis or hepatosplenomegaly. In comparison with these previously published cases, our case is different in the particularly early presentation at 3 months of age, the absence of neurologic manifestations, the presence of severe pancytopenia with an initially normal MCV, and the identification of maternal celiac disease as the underlying etiology.

In the present case, maternal vitamin B12 deficiency secondary to celiac disease resulted in severe infantile pancytopenia requiring transfusions and extensive diagnostic evaluation. Earlier recognition through antenatal screening could likely have prevented this presentation. This highlights the need for prenatal care programs to incorporate routine or targeted vitamin B12 assessment alongside folic acid and iron supplementation, particularly in high-risk populations.

In conclusion, this case illustrates that vitamin B12 is as essential as folic acid in prenatal care. Comprehensive prenatal follow-up programs that include early assessment and timely replacement of vitamin B12 can prevent severe and potentially irreversible complications in infants, improve diagnostic accuracy, and enhance both maternal and neonatal outcomes.

## Figures and Tables

**Table 1:** Initial lab results upon admission and lab results four months after admission.

Test	Result	Reference Range	Result	Reference Range	Unit
Hemoglobine	3.9	9.5–13.5	12.2	10.5–13.5	g/dL
Erythrocytes	1.24	3.10–4.50	4.95	3.70–5.30	×10^12^/L
RDW	28.8	12.1–16.2	13.2	11.8–14.2	%
Hematocrit	11.9	29.0–41.0	35.5	30–42	%
MCV	96.0	74.0–108.0	71.7	70.0–86.0	fL
MCH	31.5	25.0–35.0	24.6	23.0–31.0	pg
MCHC	32.8	30.0–36.0	34.4	32.4–35.9	g/dL
Leucocytes	6.34	6.00–17.50	11.3	6.0–17.0	×10^9^/L
Neutrophil count	1.22	1.20–5.70	5.07	1.50–8.00	×10^9^/L
Lymphocyte count	4.04	4.00–13.50	4.38	3.00–9.50	×10^9^/L
Monocyte count	0.80	0.50–1.90	0.98	0.10–1.20	×10^9^/L
Eosinophil count	0.04	0.00–0.40	0.64	0.02–0.25	×10^9^/L
Platelets	18	307–619	389	166–396	×10^9^/L
Reticulocyte count	50.6	37.0–104.0	32	24–102	×10^9^/L
Iron	267	16–128	54	50–120	µg/dL
Transferrin	2.64	1.07–3.24			g/L
ferritin	353	14–647	51	7–140	µg/L
Total bilirubin	5.5	0.1–0.7	0.26	0.20–1.20	mg/dL
conjugated bilirubin	0.4	0.1–0.3	<0.10	≤0.30	mg/dL
Unconjugated bilirubin	5.1		0.22	0.1–1.20	mg/dL
LDH	1874	163–452	293	155–286	U/L
CRP	8	<6			mg/L
Haptoglobin	<0.03	0.00–3.00			g/L
Vitamine B12			723	211–911	ng/L

## Data Availability

The original contributions presented in this study are included in the article. Further inquiries can be directed to the corresponding author.
